# Identification of different mechanisms leading to *PAX6* down-regulation as potential events contributing to the onset of Hirschsprung disease

**DOI:** 10.1038/srep21160

**Published:** 2016-02-16

**Authors:** María Valle Enguix-Riego, Ana Torroglosa, Raquel María Fernández, María José Moya-Jiménez, Juan Carlos de Agustín, Guillermo Antiñolo, Salud Borrego

**Affiliations:** 1 Department of Genetics, Reproduction and Fetal Medicine. Institute of Biomedicine of Seville (IBIS), University Hospital Virgen del Rocío/CSIC/University of Seville, Seville, 41013, Spain; 2 Centre for Biomedical Network Research on Rare Diseases (CIBERER), Seville, 41013, Spain; 3 Department of Pediatric Surgery, University Hospital Virgen del Rocío, Seville, 41013, Spain; 4 Department of Pediatric Surgery, General University Hospital Gregorio Marañon, Madrid, 28009, Spain

## Abstract

Hirschsprung disease (HSCR) is attributed to a failure of neural crest derived cells to migrate, proliferate, differentiate or survive in the bowel wall during embryonic Enteric Nervous System (ENS) development. This process requires a wide and complex variety of molecules and signaling pathways which are activated by transcription factors. In an effort to better understand the etiology of HSCR, we have designed a study to identify new transcription factors participating in different stages of the colonization process. A differential expression study has been performed on a set of transcription factors using Neurosphere-like bodies from both HSCR and control patients. Differential expression levels were found for *CDYL, MEIS1, STAT3* and *PAX6*. A significantly lower expression level for *PAX6* in HSCR patients, would suit with the finding of an over-representation of the larger tandem (AC)m(AG)n repeats within the *PAX6* promoter in HSCR patients, with the subsequent loss of protein P300 binding. Alternatively, *PAX6* is a target for DNMT3B-dependant methylation, a process already proposed as a mechanism with a role in HSCR. Such decrease in *PAX6* expression may influence in the proper function of signaling pathways involved in ENS with the confluence of additional genetic factors to the manifestation of HSCR phenotype.

Hirschsprung disease (HSCR, (OMIM 142623), the most common neurocristopathy in humans (1:5000 newborns), is characterized by the absence of enteric ganglia along variable lengths of the distal gastrointestinal tract, resulting in severe intestinal dysfunction^1^. It appears either with a familial basis or, most often, sporadically exhibiting a complex pattern of inheritance with low sex-dependent penetrance and variable expression. Such aganglionosis is attributed to a failure to fully colonize the gastrointestinal tract by enteric neural precursor crest derived cells (ENCCs) during embryogenesis. The Enteric Nervous System (ENS) development is a process finely directed by cell surface receptors and their ligands, transcription factors that regulate their expression and proteins transmitting different signals^2^. Differentiation of the ENCCs during migration is one of the key processes required to achieve the dynamic and complex circuit of neurons and glia in the course of the ENS formation^3^,^4^,^5^. This complex process is finely regulated by a large number of transcription and signaling factors and alterations throughout such processes can lead to drastic consequences as evidenced by the aganglionosis observed in HSCR.

The *RET* proto-oncogene (OMIM 164761) is the main gene associated to HSCR with differential contributions of its coding and non-coding mutations^6^,^7^. In addition, this phenotype has been associated with mutations in sets of genes involved in survival, proliferation, differentiation and migration processes, either in isolated cases or syndromic presentations of the disease which suits with the complex nature of the ENS formation^7^,^8^.

Several transcription factors are critical for the correct time course of ENS development. In humans *SOX10*, *PHOX2B* and *NKX2-1* (*TTIF1*), previously associated to some isolated or syndromic forms of HSCR, act as potential regulators of *RET* expression and *SOX10* also modulate *EDNRB* expression^9^,^10^,^11^,^12^. In enteric precursors from mice and human, *Pax3* is required for the activation of *Ret* transcription co-operatively acting with *Sox10*^13^,^14^. In addition, it has been shown that the ablation of *Zfhx1b* in mice neural crest prevents ENCCs migration beyond the proximal duodenum, and mutations in this gene are a cause of the Mowat-Wilson syndromic form of HSCR^15^.

Nevertheless, mechanisms underlying enteric precursor cell fate decisions during colonization of the bowel are relatively poorly understood. In an effort to better understand the etiology of HSCR and the signals required for a proper ENS development, we have designed a study to identify new transcription factors participating in different stages of the colonization process. With such purpose, we have performed a large-scale real-time PCR expression study of transcription factors participating in Embryonic Stem Cell networks that might be involved in ENS development, specially focusing on cell proliferation and/or differentiation process. For this study we have used human enteric precursor cells isolated from colon tissue of HSCR patients and control individuals as Neurospheres-like bodies (NLBs).

## Results

### Characterization of enteric neural precursor cells. Different expression of transcription factors in HSCR patients *versus* Control individuals

We have evaluated the gene expression profile of 48 transcription factors, all of them previously described as cell proliferation or differentiation modulators, which may be potential participants in the interactome network of enteric precursor cells during ENS development. Analysis and display of quantitative gene expression assay was positive for 14 transcription factors (Supplementary Fig. S1, Table S1 and Table S2 online), four of which showed statistically significant differential expression patterns (>2-fold change) between HSCR-NLBs and Control-NLBs (Table 1). These genes were *CDYL*, *MEIS1*, *STAT3* (up-regulated) and *PAX6* (down-regulated). Among these results, the most significant finding was the reduction by 4.67 fold of (cDNA)*PAX6* expression in HSCR-NLBs compared with Control-NLBs (p value = 0.0006). *PAX6* is a regulator required for a correct differentiation and proliferation in the signaling and formation of the Central Nervous System (CNS). Therefore, in light of these results we further focused on *PAX6* as a candidate gene in the pathogenesis of HSCR.

### *PAX6* expression was reduced in HSCR-NLBs. The expression ratio *PAX6*/*PAX6*(5a) vary within narrow limits in NLBs

**Figure 1:**
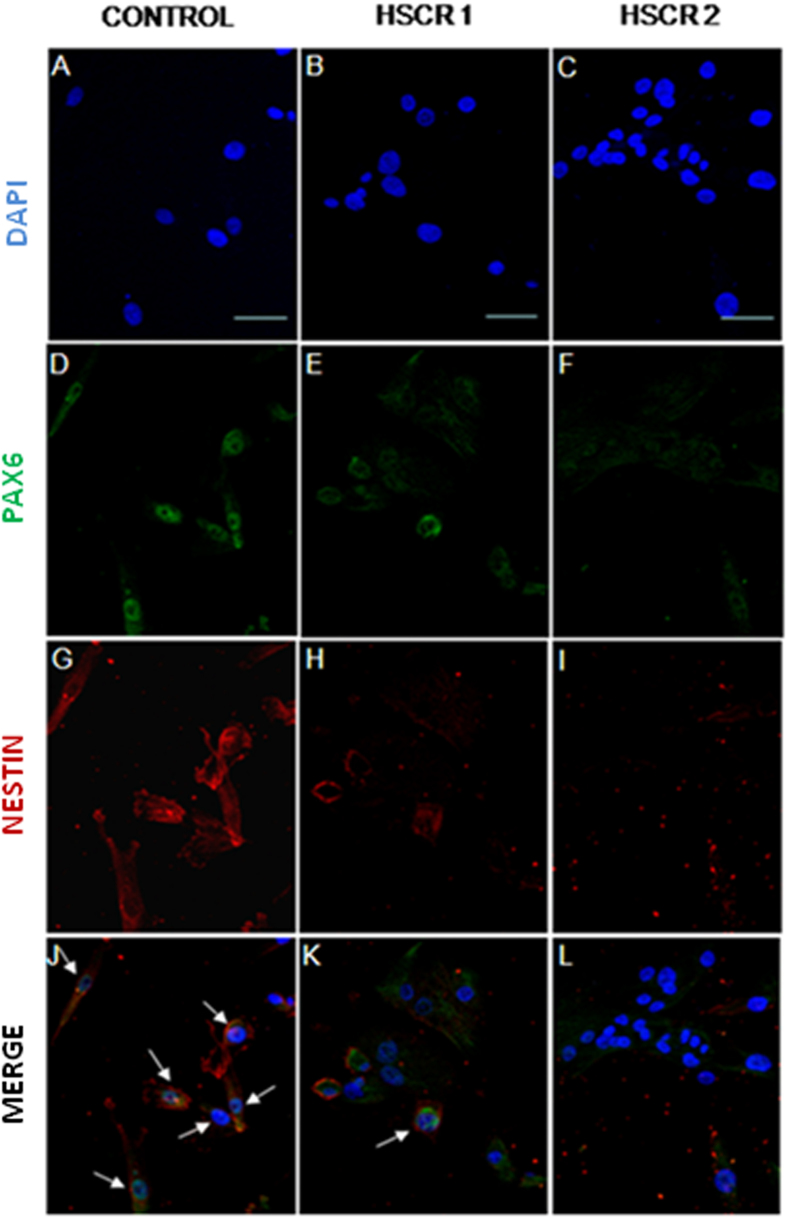
Expression of *PAX6* in HSCR-NBLs compared with control-NLBs. Confocal images of protein expression of *PAX6* in neural precursors derived from controls (**A**,**D**,**G**,**J**) and HSCR patients (**B**,**C**,**E**,**F**,**H**,**I**,**K**,**L**). Immunostaining for *PAX6* is shown in green and for NESTIN is shown in red. Cell nuclei were counterstained with 4´,6-diamidino-2-phenylindole (blue). Scale bars = 25 μM.

The reduced expression levels of (cDNA) *PAX6* in HSCR-NLBs compared to Control-NLBs were also verified by immunocytochemistry. The percentage of cells expressing *PAX6* was 36% in HSCR compared to controls, which represents a 64% of reduction in the basal values of expression (Fig. 1). It is relevant to note, that positive *PAX6* cells express a marker from neural precursor cells (NESTIN) supporting that these cells are neural precursors. We have also analyzed the conditional expression of the two major isoforms of *PAX6* in HSCR-NLBs, given that changes in the relative concentrations of these isoforms of *PAX6* [*PAX6*/*PAX6*(5a)] during development and neurogenesis, result in changes in the functions of its downstream regulated genes. However, we did not detect different expression levels between cDNA of *PAX6/PAX6 (5a)* (HSCR-NLBs Ct-*PAX6:*32.7 and Ct-*PAX6(5a):*33.16; Control-NLBs Ct-*PAX6*:23 and Ct-*PAX6(5a):*22). The (cDNA) *PAX6/PAX6*(5a) values after normalization were 1,0175 and 1,09 in HSCR and controls respectively.

### Identification of variants within *PAX6* sequence

Mutational screening of *PAX6* sequence in 196 HSCR patients, revealed no pathogenic variants, ruling out therefore the presence of deleterious mutations in this gene as a mechanism leading to HSCR (Supplementary Table S3 online).

### Significant different STR allelic and genotypic distribution at the *PAX6* P1 promoter region in HSCR *versus* controls

**Figure 2:**
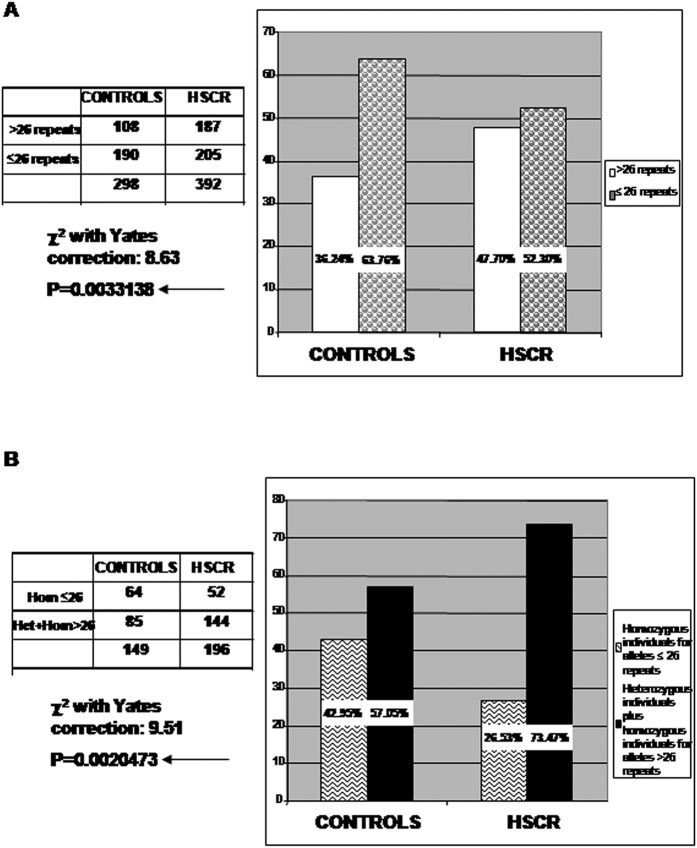
Allelic and genotypic distribution of (AC)m(AG)n repeats within the *PAX6* P1 promoter region. (**A**) Distribution of *PAX6* alleles with >26 (AC)m(AG)n repeats in HSCR *versus* controls. (**B**) Distribution of individuals carrying at least one *PAX6* allele with >26 (AC)m(AG)n repeats. Heterozygous and homozygous individuals with alleles >26 repeats were grouped.

The analysis of the allelic distribution for the (AC)m(AG)n repeat at the *PAX6* P1 promoter revealed a total of 13 different alleles for HSCR, ranging from 23 to 35 repeats, and 10 different alleles for controls, ranging from 24 to 33 repeats, the allele frequency can be found as Supplementary Fig. S2 online. In general, allelic distribution showed to be significantly different between HSCR and controls (x^2^ = 21.00, p = 0.0072). The most common allele for both groups was the one with 26 repeats, although its frequency was 11.4% lower in HSCR in comparison to controls (44.89% vs 56.3%). Moreover, when analyzing the distribution of each specific allele, we found significant under-representation of the 26 repeats allele (x^2^ = 8.98, p = 0.0027) and over-representation of the 29 repeats allele (x^2^ = 10.23, p = 0.0014) in the group of patients. These findings led us to set up a cut-off point at 26 repeats, and a detailed inspection showed that alleles with 26 or less repeats accounted for 52.29% of patients and 63.76% of controls, whereas the >26 repeats alleles accounted for 47.71% of patients and 36.24% of controls (x^2^ = 8.63, p = 0.0033) (Fig. 2A). In addition, when analyzing the genotypic distribution among both groups, we also found a significantly different distribution of homozygous individuals for ≤26 repeats alleles (HSCR: 26.53%, controls: 42.95%), heterozygous individuals (HSCR: 51.53%, controls: 41.61%) and homozygous individuals for >26 repeats alleles (HSCR: 21.94%, controls: 15.44%) (χ^2^ = 10.42, p = 0.0055). Statistical significance was maintained when analyzing the genotypic distribution after assembling heterozygous individuals and homozygous individuals with >26 repeats alleles (χ^2^ = 9.51, p = 0.0020) (Fig. 2B). These findings are concordant with an over-representation of HSCR patients carrying at least one or both alleles with >26 repeats (73.47%) in comparison with controls (57.05%).

To further investigate the possible impact of the (AC)m(AG)n differential representation at *PAX6* P1 promoter, we used *in silico* approaches aiming to analyze any differential binding of regulator molecules. We submitted different alleles containing variable repeats to TFSEARCH and, noteworthy, the analysis revealed a loss of the binding of E1A Binding Protein P300 (P300) for *PAX6* alleles containing ≥29 repeats. Furthermore, such loss was observed with different combinations of (AC) and (AG) units leading to alleles of 29 or more repeats.

**Figure 3:**
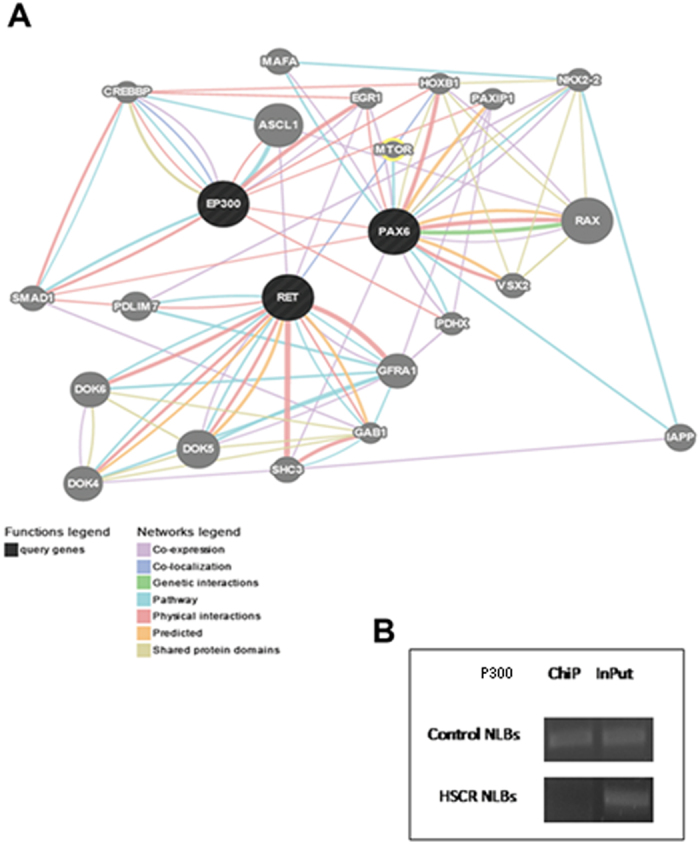
Interactions of PAX6 with P300 and other HSCR-related proteins. (**A**) Report of GeneMANIA search, which shows a physical interaction between PAX6 and P300 and indirect interactions with RET. (**B**) Amplification bands corresponding to the region (AC)m(AG)n repeat at the *PAX6* P1 promoter, isolated from the inmunoprecipitated samples with P300 antibody (ChiP) from Control- NLBs (up) and HSCR-NLBs (down). The amplification band of the coresponding InPut samples were considered as positive controls.

Gene-Mania and Var-Elect tools were then used to analyze interactions between PAX6 and P300, and predictions revealed a direct physical interaction. Moreover, both proteins were found to be indirectly related to RET and other proteins encoded by genes previously associated to HSCR (Fig. 3A).

### *PAX6* is a target of P300

To further investigate the role of P300 in the interplay of HSCR and its relation with *PAX6*, we checked first for the expression of P300 in HSCR and control NLBs obtaining positive expression (Supplementary Table S4). In order to study the physical interaction between P300 and *PAX6* which *in silico* analysis suggested, ChIP-PCR analysis was performed using anti-P300 antibody in human NLBs from one control and one HSCR patient. This assay confirmed the binding of P300 to the region encompasses the (AC)m(AG)n tandem in control-NLBs. However, the band was dramatically reduced in the HSCR patient (Fig. 3B). DNA isolated from both samples was afterward genotyped; the HSCR patient carried out both alleles with 28 repeats and the control harbored alleles with different number of repeats (26/27).

According to data from the ALGGEN-PROMO bioinformatics tool, the specific binding sites for P300 were identified just flanking the repeating dinucleotide within *PAX6* sequence. The ability of P300 to interact with *PAX6* might be affected by the distance between these regions. The increasing number of repeats may influence the conformation of that particular sequence modifying the access of the protein to its binding site, thus we found differences between patient and control related to P300 binding affinity depending on the number of repeats.

### *PAX6* is a target of the Methyltransferase DNMT3B

**Figure 4:**
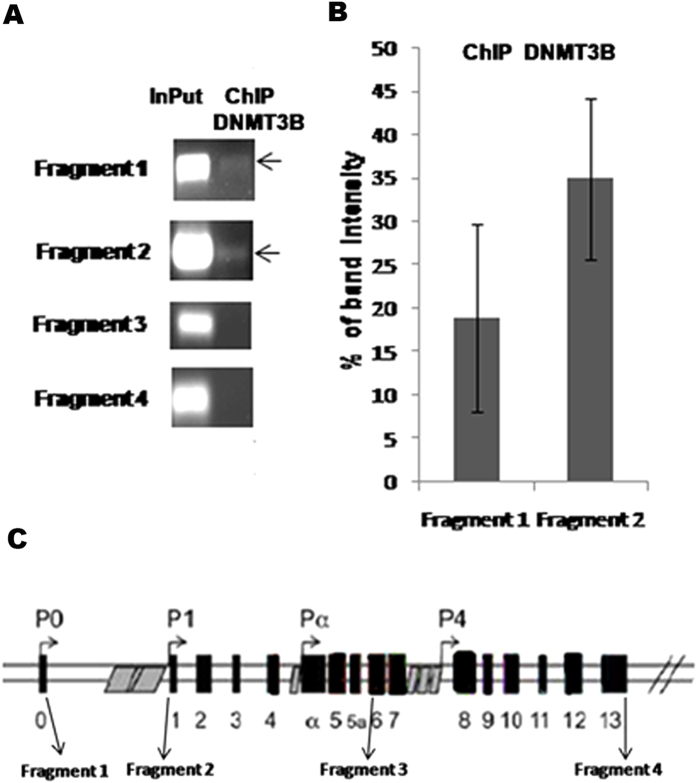
Identification of *Pax6* as a target of Dnmt3b methylation by ChiP-PCR. (**A**) Amplification bands corresponding to a selection of different regions of *Pax6*, isolated from the immunoprecipitated samples with Dnmt3b antibody. Amplification was observed for fragments 1 and 2. (**B**) Graph showing the relative mean intensity values of the corresponding amplification bands after normalization with the InPut. (**C**) Scheme of the *Pax6* gene showing precise regions for analized fragments.

The methyltransferase DNMT3B is essential for the *de novo* methylation process that occurs during neurogenesis in the embryonic development. As previously reported by Torroglosa *et al.*, HSCR-NLBs displayed lower DNA methylation and less expression of (cDNA) *DNMT3B* and (cDNA) *PAX6* compared with Control-NLBs^16^. Based on these results, we aimed to elucidate if *PAX6* is a direct target of DNMT3B and therefore its expression might be regulated by methylation. For this purpose, ChiP-PCR assay was performed in NLBs from mice. Samples after ChIP assay with anti-Dnmt3b antibody were amplified by PCR. Two out of four *Pax6* studied regions were detected in ChiP samples (Fig. 4). These fragments correspond to regions that show a high degree of homology (59,75% and 85,18% respectively) with humans and they are also within the *PAX6* human CpG islands called *CpG35* and *CpG464* annotated by the UCSC Genome Browser database (http://genome.ucsc.edu/) (Supplementary Fig. S3 online).

## Discussion

The ENS arises from neural crest-derived cells with multipotency and migratory capabilities leading to the formation of a complex network of enteric neurons and glial cells that colonize the gastrointestinal tract. This complex process is carefully regulated by interacting signals and transcription factors that confer cell characteristics in each moment of the ENS development, and it is accepted that failures in this process are responsible for HSCR pathogenesis. Understanding underlying transcriptional programs required for enteric precursors normal development might help us to identify new genes and pathways involved in HSCR pathogenesis. With this aim, through the expression study performed among HSCR and controls NLBs, four new transcription factors (*CDYL, MEIS1, STAT3* and *PAX6)* that might be involved in human enteric precursor cells interactome networks have been identified. It comes as no surprise that all transcription factors found to be deregulated in ENS precursors from HSCR, are implicated in the transition of proliferation/differentiation state, a critical process for the correct ENS formation. *CDYL* is a chromodomain-containing transcriptional co-repressor ubiquitously expressed. A study conducted in mice suggests that during neural development, *Cdyl* might be inhibiting the neuronal differentiation of induced pluripotent stem cells (iPS)^17^,^18^. *MEIS1* is a conserved transcription factor that specifically acts as a direct molecular regulator of *PAX6* expression during vertebrate’s development^19^,^20^. *STAT3* is part of the JAK-STAT signaling pathway and plays an important role in the coordination of the succession of steps along the cell cycle and the differentiation process during neural crest cell specification^21^,^22^. Finally, we also obtained deregulated expression levels for the transcription factor *PAX6.* This gene is a highly conserved transcription factor belonging to the family of the so-called “paired-box” with functions in the eye, central nervous system and pancreas development. *PAX6* is a regulator of the neural precursor proliferation and differentiation which acts by modulating the expression of different downstream effectors. Its main role is the generation of new neurons from stem cells and neural progenitors during the initial stages of the CNS development^23^,^24^,^25^. In the developing brain, *PAX6* initially modify the proliferation of progenitor cells and later neural differentiation in a highly context-dependent manner. This changing role might be due to the relative balance of the two major isoforms *PAX6* and *PAX6*(5a) expression levels during development^26^. Nevertheless, the enteric precursor cells showed no differences between expression levels of both (cDNA)*PAX6* isoforms, leading to the conclusion that the ratio *PAX6*/*PAX6*(5a) does not seem to be essential for the enteric precursor decision (proliferation/differentiation).

Deregulated expression levels for *PAX6* confirmed data previously obtained by Torroglosa *et al.*, 2014, which prompted us to explore additional and different mechanisms underlying the *PAX6* lower expression observed in HSCR-NLBs.

Several human diseases are caused by non-coding variations at regulatory regions of the respective genes, whose mechanism consists of the disruption of a proper regulation of the gene expression. In Friedreich ataxia (FRDA), for instance, GAA-repeat expansion in the first intron of *FRDA* gene results in decreased levels of frataxin due to inhibition of transcriptional elongation. Another example is FRAXA, where the expansion of a CGG-repeat in the 5′UTR of the *FMR1* gene, leads to hypermethylation of the CpG Island, transcriptional silencing and loss of the protein product.

*PAX6* expression is regulated by alternate usage of two promoters (P0 and P1) which are differentially regulated in a tissue-specific manner^27^ and its activation is positively related to *PAX6* transcripts expression. P1 promoter contains distinct *cis* elements and several potential binding sites for transcription factors involved in tissue-specific expression in the eye, central nervous system and pancreas^28^,^29^,^30^. One of these elements is a polymorphic (AC)m(AG)n repeat located about 1 kb from the transcription start site which influences the transcriptional promoter efficiency in brain^31^,^32^. The analysis of the allelic and genotypic distribution of this repetitive element in HSCR patients *versus* control individuals, showed an over-representation of >26 repeats alleles in HSCR patients in comparison with control subjects. *In silico* analyses revealed the existence of two putative binding sites, just flanking the repetitive sequence, recognized by P300. This protein is a histone acetyltransferase essential in the processes of cell proliferation and differentiation regulating transcription via chromatin remodeling. Subsequently, ChIP analyses revealed the existence of that physical interaction between *PAX6* and the protein P300 in enteric precursors. However, the ability of P300 to bind its target sequence was dramatically reduced when the number of repeats was increased. Changes in the repeat number might alter the distance between P300 binding sites, thus modifying this protein binding affinity. This specific DNA region within *PAX6* promoter sequence must undergo structural changes to either facilitate or hinder the accessibility to P300. The increase of repeats may also have influence on the conformation of the sequence and generate different structures for other proteins recognition. This mechanism might contribute to a lower expression of *PAX6* and, probably leads to a decrease in regulation of the signaling pathways related to the ENS development in which this protein is involved. Regarding to the complex genetic basis of HSCR disease, we propose this mechanism as a necessary event contributing to *PAX6* down-regulation observed in patients, even though other conditions are required to completely elucidate this finding.

Epigenetic programs are essentials to drive the transcriptional profiles required for a normal neural development. As described by Torroglosa *et al.*, an aberrant methylation status in HSCR-NLBs, was proposed as a mechanism responsible for incorrect gene expression patterns in these patients^16^. Regulation of gene expression through methylation is one of several ways to modulate many cellular processes during development, particularly in neurogenesis^33^. Mammalian postnatal neurons express at high levels DNA methyltransferases and DNA methylation along these cells is extremely dynamic^34^.

Furthermore, it has been shown that *PAX6* expression levels may be disturbed by methylation^35^,^36^. With the aim of studying if *PAX6* is a target of DNMT3B and therefore susceptible of being regulated by methylation, we performed a new ChIP-PCR analysis. *Pax6* was shown to be a target for Dnmt3b, as well, as we identified two specific regions of this gene to where Dnmt3b is attached. Such binding sites are within CpG-rich regions in the human *PAX6* sequence.

We hypothesize that enteric precursors from HSCR-NLBs seem to be non-responsive to external signals that lead to the correct neuronal differentiation, so that this event cannot occur at the right time, regardless of the balance between *PAX6* isoforms as it has been discussed previously.

We have evaluated the gene expression profile of transcription factors which may be potential participants in the gene regulatory network of enteric precursor cells in the context of ENS development and HSCR. Findings of reduced *PAX6* expression levels in HSCR-NLBs supports that *PAX6* operates and regulates transcriptional network during the ENS development. We report the first association study of the transcription factor *PAX6* with HSCR and that its low expression levels may result in an aberrant neurogenesis, which is directly related with manifestation of HSCR phenotype.

## Methods

### Generation of ENS NLBs

ENS progenitor cells were obtained from human postnatal tissues of ganglionic gut of six sporadic non-related patients diagnosed with isolated HSCR (L-HSCR:S-HSCR = 2:4; male: female = 3:3) and six patients of other gastrointestinal disorders undergoing gut resection surgery at Hospital were used as controls (4 males and 2 females). For both HSCR patients and control individuals, age range was between 3 months and 3 years.

The extraction of ENS progenitor cells from ganglionic bowels in mice was carried out in the same way as in humans, as previously described in Torroglosa *et al.*^16^.

Written informed consent for surgery, clinical and molecular genetic studies was obtained from all participants. The study was approved by the Ethics Committee for clinical research of the University Hospital Virgen del Rocío (Seville, Spain) and complies with the tenets of the declaration of Helsinki.

All procedures involving mice were performed in accordance with European Union guidelines (2010/63/EU) and Spanish law (R.D. 53/2013 BOE 34/11370-420, 2013) concerning the care and use of laboratory animals and were approved by the Animal Experimentation Ethics Committee (EAEC/IEC) of University Hospital Virgen del Rocío / Institute of Biomedicine of Seville (IBIS).

### Gene Expression Study by Quantitative Real-Time PCR (qRT-PCR)

A differential expression study in cultures of NLBs has been performed in a set of transcription factors implicated in the regulatory networks of embryonic stem cells (Supplementary Table S5 online). Purification and synthesis of cDNA were performed using the protocol provided by μMACS mRNA isolation Kit and μMACS cDNA Synthesis Kit in a thermo MAKSTM Separator (MACS Miltenyi Biotec, Germany). Expression studies were carried out in an Applied Biosystems 7900HT system (Life Technologies, USA) through the TaqMan® Human Transcriptional Regulatory Network in Embryonic Stem Cell Array Plate (Life Technologies, USA) and SYBR Green method (Bio-Rad, USA). Amounts of 1000 ng (10 ng/μL) of cDNA (converted from total RNA) were used per fill reservoir for the amplification reactions through the Taqman Low Density Array. For the amplification studies performed with SYBR, 2000 ng (100 ng/μL) of cDNA (converted from total RNA) were added per well, following manufacturer recommendations.

Analysis was performed using the RQ Manager Software (Life Technologies, USA) based on the comparative Ct (ΔΔCt) method. *GAPDH* or *B-ACTIN* was used as endogenous control respectively. Following the software recommendations, the upper limit of the cycle threshold (Ct) was set at 32 for the TaqMan Array Gene Signature Plates and 35 for the SYBR Green assay. Positive expression was exclusively considered when Ct values were lower than values described above.

### Statistical analyses for gene expression study

Data are presented as the mean ± SEM (Standard Error Mean) of values obtained from at least three experiments. Comparisons between values obtained in Control-NLBs and HSCR-NLBs were analyzed using the Student´s t test. Differences were considered significant when *p value* < 0.05.

### Immunocytochemistry

For immunocytochemical studies, cells derived from NLBs were seeded onto coverslips fibronectin-poly D lysine coated and fixed with 4% (wt/vol) paraformaldehyde in 0.1 M PBS. Then, they were incubated for one hour in 2.5% (wt/vol) bovine serum albumin (BSA) in PBS and with primary and secondary antibodies. After washing, the coverslips were mounted on slides with Fluoro-Gel (EMS, Hatfield, PA, USA) and fluorescent signals were detected using a Leica Spectra confocal microscope. The primary antibodies used were anti-*PAX6* (rabbit polyclonal, 1:20) and anti-NESTIN (goat polyclonal, 1:400) (Santa Cruz Biotechnology, Inc). The secondary antibodies used were anti-rabbit IgG labeled with Cy2 (1:200) and anti-goat IgG labeled with Cy5 (1:200) (Jackson Immuno Research Laboratories, Inc.). The nuclei of cells were counterstained with DAPI. For the quantification, ten images were taken per condition in which at least a number of 700 cells were counted.

### Molecular Analysis of *PAX6*

For molecular studies we included a total of 196 Spanish HSCR patients (124 males/72 females) comprising sporadic and apparently isolated cases. A total of 158 cases were short segment forms (S-HSCR), 31 were long segment forms (L-HSCR) and 7 presented with total colonic aganglionosis (TCA). In addition, we also analyzed a group of 150 normal control individuals comprising unselected, unrelated, race-, age-, and sex-matched individuals. This study was approved by our Ethics Committee for clinical research and conformed to the tenets of the declaration of Helsinki. Fully written informed consent for molecular genetic studies was obtained from all the participants. Genomic DNA was extracted using standard protocols from peripheral blood leukocytes of all individuals included in the study.

The mutational screening of the complete coding sequence of *PAX6* (NM-001127612) was performed by direct sequencing using an ABI Prism 3730 Genetic Analyzer and SeqScape v2.5 software (Life Technologies, Carlsbad, CA; conditions available on request).

On the other hand, genotyping of the (AC)m(AG)n STR, located approximately 1 kb upstream of the transcription initiation site and associated with promoter P1 of *PAX6*, was performed by fluorescent PCR and subsequent analysis in the ABI Prism 3730 Genetic Analyzer with the Genemapper software (Life Technologies, Carlsbad, CA; conditions available on request). Statistical analysis of the allelic and genotypic distribution in HSCR patients *versus* controls was performed using Pearson or Chi Squared analysis, with statistical significance set at p < 0.05. Data were analyzed employing the Statistical Package for Social Sciences (SPSS) Version 14.0 for Windows.

To perform *in silico* predictions of transcription factors binding sites we have used TFSEARCH (http://diyhpl.us/~bryan/irc/protocol-online/protocol-cache/TFSEARCH.html). ALGGEN-PROMO (http://alggen.lsi.upc.es/cgi-bin/promo_v3/promo/promoinit.cgi?dirDB =  TF_8.3), was used to predict transcription factor binding sites in DNA sequences. Putative interactions between different genes and their participation in signaling pathways were analyzed with both Gene-Mania (http://www.genemania.org) and Var-Elect (http://www.genecards.org/?path =  /Search/VarElect).

### Chromatin Immunoprecipitation Assay (ChIP) and PCR

Enteric NLBs from mice and human were cross-linked with formaldehyde 1%. Cell breakage from NLBs was performed in lysis buffer (SDS, Lysis buffer-ChIP Assay, Kit Millipore Corporation, USA) and sonicated in a Bioruptor® system (Diagenode Inc., USA). Immunoprecipitation was performed with P300 and Dnmt3b antibody (Abcam, UK) following the instructions of ChIP Assay Kit (Millipore Corporation, USA) in two different assays. Amplification of the region (AC)m(AG)n STR within the *PAX6* promoter and four different regions that cover the full gene (*Pax6*, NM_013627) (Supplementary Table S6 and S7 online) were performed in each input and inmunoprecipitated sample. Finally, the amplification products were separated in 2% agarose gel by electrophoresis.

## Additional Information

**How to cite this article**: Enguix-Riego, M. V. *et al.* Identification of different mechanisms leading to *PAX6* down-regulation as potential events contributing to the onset of Hirschsprung disease. *Sci. Rep.* **6**, 21160; doi: 10.1038/srep21160 (2016).

## Supplementary Material

Supplementary Information

## Figures and Tables

**Table 1:** Genes with a significantly different expression in HSCR-NLBs *versus* control-NLBs.

Genes	Expression	P value
CDYL	Up-regulated	0.02
*MEIS1*	Up-regulated	0.008
*STAT3*	Up-regulated	0.006
*PAX6*	Down-regulated	0.0006
