# Supplementary material for: Identification of different mechanisms leading to PAX6 down-regulation as potential events contributing to the onset of Hirschsprung disease
Source: Sci Rep. 2016 Feb 16;6:21160. doi: 10.1038/srep21160 (PMC4754768; doi:10.1038/srep21160)
Supplement: Supplementary Information [file srep21160-s1.pdf]

TITLE: Identification of different mechanisms leading to *PAX6* down-regulation, as potential events contributing to the onset of Hirschsprung disease

AUTHOR: María Valle Enguix-Riego[1,2,#], Ana Torroglosa[1,2,#], Raquel María Fernández[1,2], María José Moya-Jiménez[3], Juan Carlos de Agustín[4], Guillermo Antiñolo[1,2], Salud Borrego[1,2]*

# These authors contributed equally

AUTHOR INSTITUTIONAL AFFILIATIONS:

[1]Department of Genetics, Reproduction and Fetal Medicine. Institute of Biomedicine of Seville (IBIS), University Hospital Virgen del Rocío/CSIC/University of Seville, Seville, 41013, Spain.

[2] Centre for Biomedical Network Research on Rare Diseases (CIBERER), Seville, 41013, Spain.

[3] Department of Pediatric Surgery, University Hospital Virgen del Rocío, Seville, 41013, Spain.

[4]Department of Pediatric Surgery, General University Hospital Gregorio Marañon, Madrid, 28009, Spain.

***CORRESPONDING AUTHOR:**

Salud Borrego, MD, PhD.

Department of Genetics, Reproduction and Fetal Medicine.

University Hospital Virgen del Rocío.

Avda. Manuel Siurot s/n. 41013 Seville, Spain

Voice: + 34 955 012780, Fax: + 34 955 012779

e-mail: salud.borrego.sspa@juntadeandalucia.es

Figure S1

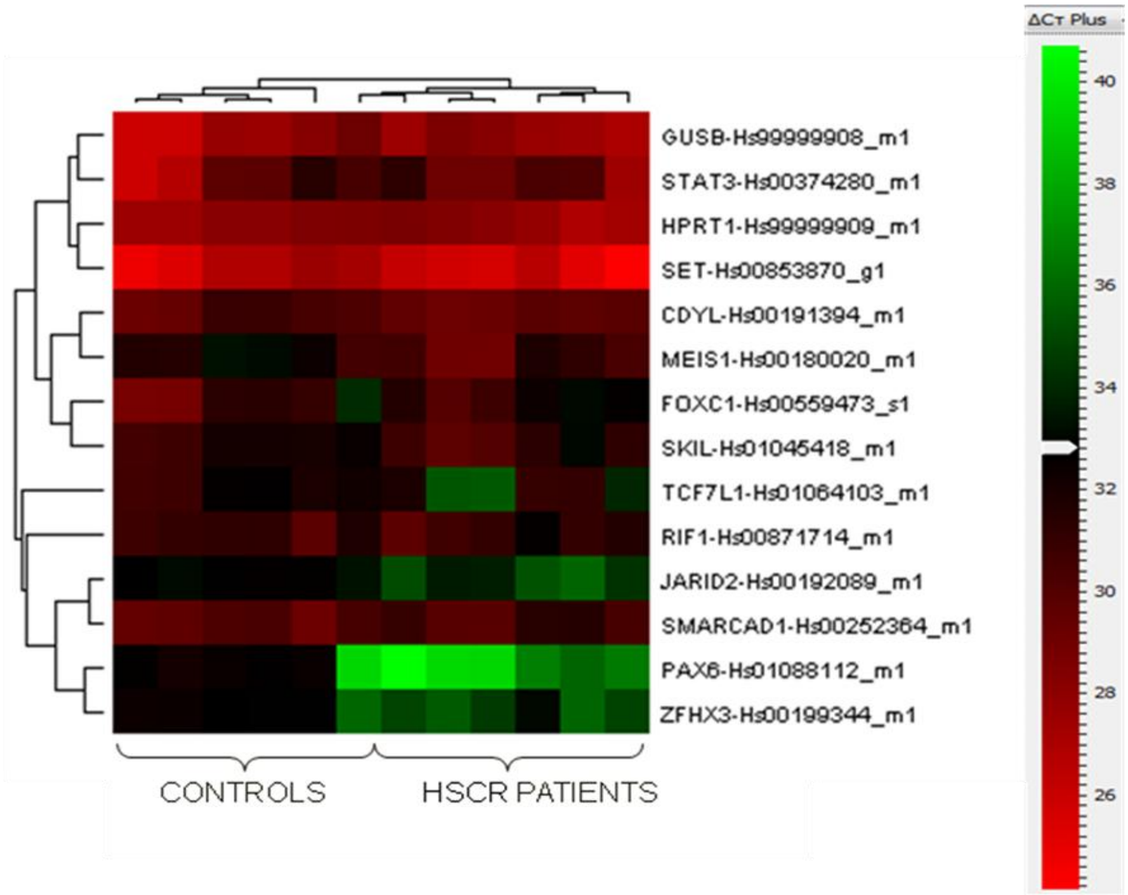

Figure S1. Graphical representation of Transcription Factors expression in human NLBs from controls and HSCR patients.

The Heat map was generated using DataAssist v3.0 software (Life Technologies) and it represents the messenger RNA expression levels of 14 transcription factors in NLBs from six controls and six HSCR patients. The expression level is set as the CT values from each sample for each assay. Genes were hierarchically clustered by Pearson correlation coefficient using average linkage. The color scale, representing ΔCt, is shown on the right side. Ct, cycle threshold; HSCR, Hirschsprung disease; NLBs, neurosphere-like bodies

Figure S2

(AC)m(AG)n	Allele count (%)	
**Repeats**	**CONTROL** (n=298)	**HSCR** (n=392)
23	0	1 (0,255%)
24	4 (1,34%)	3 (0,765%)
25	17 (5,074%)	25 (6,377%)
26	168 (56,37%)	176 (44,89%)
27	35 (11,74%)	58 (14,795%)
28	32 (10,73%)	45 (11,479%)
29	14 (4,69%)	45 (11,479%)
30	14 (4,69%)	24 (6,122%)
31	11(3,69%)	9 (2,295%)
32	2 (0,67%)	1 (0,255%)
33	1(0,33%)	1 (0,255%)
34		3 (0,765%)
35		1(0,255%)

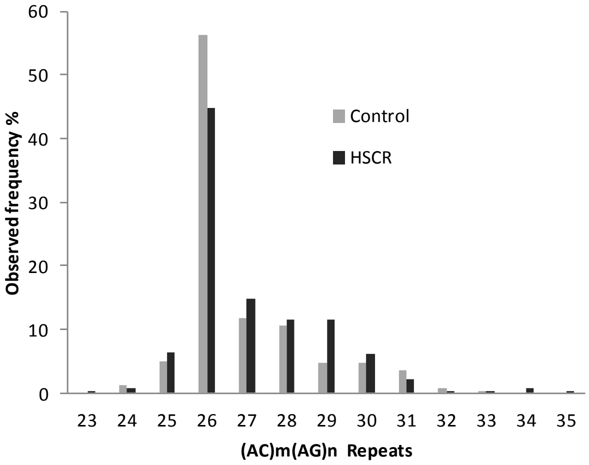

Figure S2: Allelic frequencies of *PAX6* P1 promoter dinucleotide repeats in HSCR patients and control subjects

Figure S3

Fragment 1

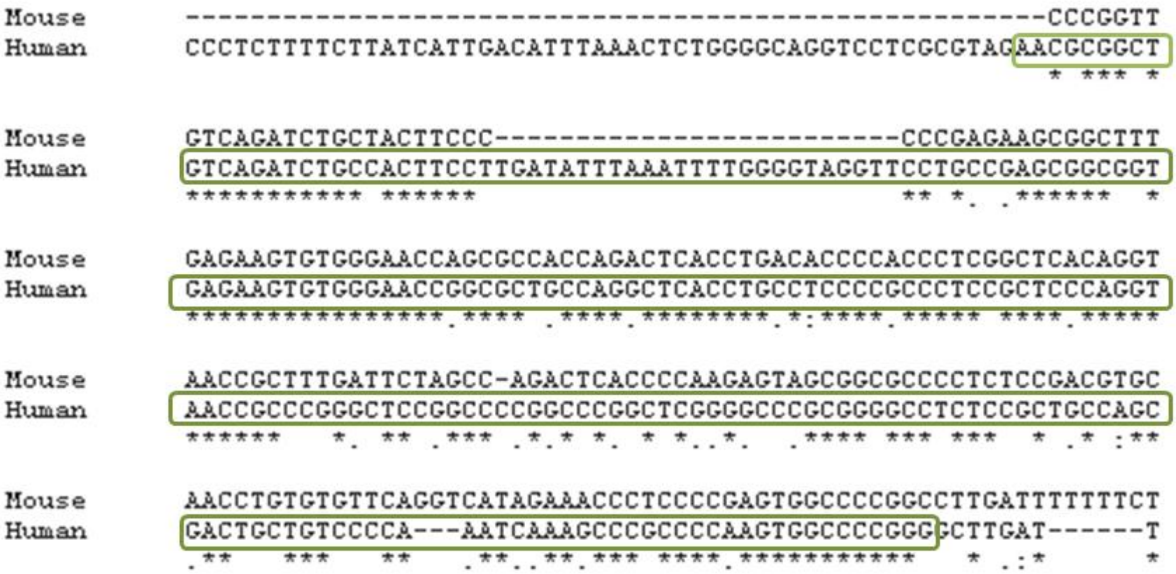

Fragment 2

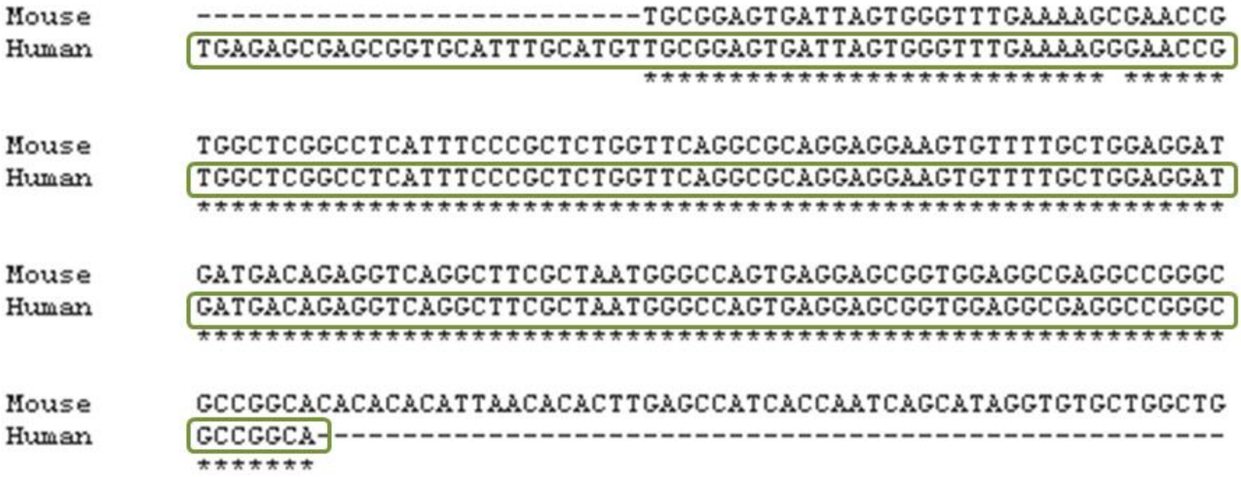

Figure S3. Two regions of the *Pax6* sequence were targets of Dnmt3B methylation in mouse. The fragments shown in the figure correspond to regions in mouse with a high homology degree in humans and they also encompass predicted CpG islands (green).

Table S1. Transcription Factors expression normalized with the endogenous control

GENE NAME	ΔCT CONTROL	ΔCT HSCR
CDYL	7,6	7,1
FOXC1	8,2	9,6
GUSB	5	5,3
HPRT1	5,7	5,5
JARID2	10,6	12
MEIS1	9,4	7,8
PAX6	11,8	15
RIF1	8,4	8,8
SET	3,8	3,2
SKIL	8,7	8,6
SMARCAD1	8	8
STAT3	7	6´5
TCF7L1	9,4	10
ZFHX3	11	12,7

Table S2. Transcription Factors Ct values in Control and HSCR NLBs

	**Ct Controls**	**Ct HSCR Patients**
CDYL	29	32
FOXC1	30	34
GUSB	26	30
HPRT1	27	30
JARID2	31	37
MEIS1	31	32
PAX6	30	39
RIF1	30	33
SET	25	29
SKIL	30	33
SMARCAD1	30	33
STAT3	28	31
TCF7L1	31	35
ZFHX3	31	37

Table S3 : Identified *PAX6* variants in HSCR patients

Nucleotide Change	Amino Acid Change	Reference
c.-6-176C>T		rs6058885
c.142+117G>A		-
c.142+79G>A		rs181671547
c. 142+36C>A		-
c.432+75G>A		-
c.432+76T>A		-
c.654+54A>G		rs4911108
c.654+30T>C		rs73258152
c.654+111G>A		rs79368590
c.654+129A>G		rs6057645
c. 922-123G>A		rs1474738
c.922-55C>A		-
c.922-57G>T		-
c.922-8C>T		rs200508870
c.1066-113G>A		-
c.1066-135G>T		-
c.1066+653G>A		rs993419
c.1066-55G>T		-
c.1252+13T>G		rs910085
c.1252-70G>T		-
c.1252-59G>C		-
c.1297+30C>T		rs875041
c. 1377+126C>T		rs6058890
c.1377+60C>T		rs192769774
c.1378-113C>A		rs6057648
c.1572T>C	Cys524Cys	rs6058891
c.1759-5C>G		rs1997797
c.1759+122C>A		-
c.1872C>T	Tyr624Tyr	-
c.1881C>T	Asp627Asp	rs17123657

Table S4. P300 expression in HSCR and Control NLBs

	Ct average HSCR	Ct average control
P300	32,4	25,0
Actin	23,3	15,2
	Ctp300-Ct Actin	Ctp300-Ct Actin
Normalization	9,1	9,8
Fold Change	1,6	

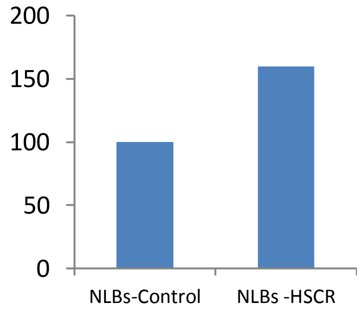

P300 Primers sequence used for the expression study:

LEFT PRIMER (5’-3’): GGCTCCAGGACAGTCAAAGA
RIGHT PRIMER(5’-3’) : TCCATGGGGCTCTTCACAAT

Table S5: Genes included in the "TaqMan® Human Transcriptional Regulatory Network in Embryonic Stem Cell Array Plate" (Life Technologies, USA).

18S	GAPDH	HPRT1	GUSB	CALB1	CDX2
CDYL	EOMES	ESX1	FOXC1	FOXD3	GATA4
GATA6	GBX2	GJD2	GRIN1	GSX2	HAND1
HESX1	HNF4A	HOXB1	ISL1	JARID2	LHX5
MEIS1	MYF5	MYST3	NANOG	NEUROD1	NEUROG1
ORC3L	OTX1	PAX6	POU5F1	REST	RFX4
RIF1	SALL1	SET	SIX3	SKIL	SMARCAD1
SOX2	STAT3	TCF7L1	TRIM24	ZFHX3	ZIC3

Table S6: Primers used for *PAX6* P1 promoter fragment amplification in ChiP-PCR performed in NLBs from HSCR patient and control using P300 specific antibody.

Forward (5'-3')	Reverse (5'-3')	Size (bp)
CACGCTGACATACCGACATC	AGGGGAGATAAGAGGGGAGG	151

Table S7: Primers used for *Pax6* fragments amplification in ChiP-PCR performed in NLBs from mice using Dnmt3b specific antibody.

Fragment	Forward (5'-3')	Reverse (5'-3')	Size (bp)
1	CCCGGTTGTCAGATCTGCTA	ATCCGCAGCAACTCCTCTAC	271
2	TGCGGAGTGATTAGTGGGTT	CGGCTGGAGAGTGAGAGATA	255
3	TGCATATAATTGAACCTGGGACA	TCGAAGACACACTCTACCTTTT	157
4	GGTGCTGGACAATGAAAACG	CTTGGCTTACTCCCTCCGAT	202
